# Follistatin‐Like 3 Enhances the Function of Endothelial Cells Derived from Pluripotent Stem Cells by Facilitating β‐Catenin Nuclear Translocation Through Inhibition of Glycogen Synthase Kinase‐3β Activity

**DOI:** 10.1002/stem.2820

**Published:** 2018-04-10

**Authors:** Sophia Kelaini, Marta Vilà‐González, Rachel Caines, David Campbell, Magdalini Eleftheriadou, Marianna Tsifaki, Corey Magee, Amy Cochrane, Karla O'neill, Chunbo Yang, Alan W. Stitt, Lingfang Zeng, David J. Grieve, Andriana Margariti

**Affiliations:** ^1^ Centre for Experimental Medicine, Queen's University Belfast Belfast United Kingdom; ^2^ Cardiovascular Division, King's College London London United Kingdom

**Keywords:** Induced pluripotent stem‐cells, Functional endothelial cells, Vascular disease, Angiogenesis, Follistatin‐like 3, Cell reprogramming

## Abstract

The fight against vascular disease requires functional endothelial cells (ECs) which could be provided by differentiation of induced Pluripotent Stem Cells (iPS Cells) in great numbers for use in the clinic. However, the great promise of the generated ECs (iPS‐ECs) in therapy is often restricted due to the challenge in iPS‐ECs preserving their phenotype and function. We identified that Follistatin‐Like 3 (FSTL3) is highly expressed in iPS‐ECs, and, as such, we sought to clarify its possible role in retaining and improving iPS‐ECs function and phenotype, which are crucial in increasing the cells’ potential as a therapeutic tool. We overexpressed *FSTL3* in iPS‐ECs and found that FSTL3 could induce and enhance endothelial features by facilitating β‐catenin nuclear translocation through inhibition of glycogen synthase kinase‐3β activity and induction of Endothelin‐1. The angiogenic potential of FSTL3 was also confirmed both in vitro and in vivo. When iPS‐ECs overexpressing *FSTL3* were subcutaneously injected in in vivo angiogenic model or intramuscularly injected in a hind limb ischemia NOD.CB17‐Prkdcscid/NcrCrl SCID mice model, FSTL3 significantly induced angiogenesis and blood flow recovery, respectively. This study, for the first time, demonstrates that FSTL3 can greatly enhance the function and maturity of iPS‐ECs. It advances our understanding of iPS‐ECs and identifies a novel pathway that can be applied in cell therapy. These findings could therefore help improve efficiency and generation of therapeutically relevant numbers of ECs for use in patient‐specific cell‐based therapies. In addition, it can be particularly useful toward the treatment of vascular diseases instigated by EC dysfunction. Stem Cells *2018;36:1033–1044*

**Significance Statement**

Endothelial cell (EC) reprograming is a highly promising regenerative strategy to develop novel therapies to improve vascular function in patients with vascular and ischemic diseases. Understanding key factors to facilitate maintenance of EC generation and function is important as we all move towards cell therapy. The present study has elucidated, for the first time, the remarkable role of follistatin like 3 in improving the function and neovascularization potential in ECs derived from induced pluripotent stem cells both in vitro and in vivo, by facilitating β‐catenin nuclear translocation, inhibition of glycogen synthase kinase‐3β activity and Endothelin‐1 induction.

## Introduction

Vascular diseases are some of the leading causes of mortality worldwide 1 with compromised endothelial integrity being at the core of their initiation and progression 2. Vascular endothelial cells (ECs) regulate vascular tone and growth, permeability, interaction with platelets and leukocytes, and maintenance of vascular homeostasis 3. As a result, any adverse alterations in endothelial physiology increase the risk for vasodegenerative pathology and tissue nonperfusion 4. However, the restoration of the luminal endothelium is a long and slow process and there are no efficacious drug‐ or gene‐based therapies to promote this process. As a result, the repair and regeneration of functional and healthy ECs in damaged tissues remains an important research focus in regenerative medicine 5.

In recent years, efforts toward EC restoration has shifted focus to stem cell‐based approaches that include isolation, expansion, and subsequent differentiation of endothelial progenitor cells 6 or pluripotent ESCs 7 for use as therapeutic tools. However, delivery of such cells to repair of damaged vasculature has encountered many restrictions, including availability of suitable and efficacious cells for therapy 8. As a result, the capability to derive ECs [induced pluripotent stem (iPS)‐ECs] from iPS Cells holds great potential for the treatment of vascular disease 9. Even though the field is now rapidly moving toward EC generation from iPS Cells for use in cell based therapies, the main limitation of generating and maintaining functional ECs with a stable phenotype still remains.

To uncover the full potential of iPS‐ECs and their therapeutic efficacy, understanding the complex mechanisms controlling these cells is essential. To start uncovering pivotal mechanisms involved in iPS‐ECs, we found that Follistatin‐Like 3 (FSTL3) was highly expressed in these cells. This led us to hypothesize that FSTL3 may play a crucial role in enhancing iPS‐ECs generation in terms of marker expression as well as function and angiogenic potential.

Follistatin is a secreted protein that in humans is encoded by the FST gene 10 and contains three conserved follistatin domains (FSDs) 11. It is a glycosylated single‐chain protein, originally observed in ovarian follicular fluid that reduces follicle‐stimulating hormone secretion 12. Follistatin‐like (FSTL) proteins contain a minimum of one FSD and have heterogeneous, but partly characterized, functions 11. Like follistatin, FSTL3, also known as FST‐related gene or Follistatin‐related protein (FSRP) 13, binds and neutralizes activin, and other transforming growth factor‐beta (TGF‐β) ligands 14. FSTL3 is highly homologous to follistatin, containing two FSDs 15. Even though FSTL3 is secreted from cells where it is highly expressed, there is indication that FSTL3 also remains intracellular 15.

Follistatin family members are multifunctional and essential in tissue restoration and repair and they have been implicated in EC differentiation 16, angiogenesis 17, and development of vasculature 18. However, the role of FSTL3 in these processes is largely unknown.

The aim of this study was therefore to elucidate the underlying mechanisms which are governed by FSTL3 in iPS‐ECs, and might drive these cells toward a more stable and functional stage. Our findings demonstrate that FSTL3 is regulated by KLF4 during iPS‐ECs generation and has a key role in improving function in iPS‐ECs. In particular, FSTL3 enhances the expression of EC markers by increasing β‐catenin protein translocation to the nucleus through inhibiting glycogen synthase kinase‐3β (GSK3β) activity and induction of Endothelin‐1. Importantly, these results were confirmed in vivo when iPS‐ECs overexpressing *FSTL3* significantly improved angiogenesis and neovascularization and blood flow recovery in the hind limb ischemic model.

## Materials and Methods

Cell culture media, serum, and cell culture supplements were purchased from ATCC, USA, Merck Millipore, USA, LONZA, Switzerland and Thermo Fisher Scientific, USA. Antibodies against VE‐Cadherin (CD144) (ab33168 and STJ96234), VEGFR (ab9530), GAPDH (ab8245), OCT‐4 (ab19857 and STJ72238), KLF4 (ab72543) eNOS (ab76198), and FSTL3 (STJ112317) were purchased from Abcam, UK or St John's Laboratory, UK. Antibodies against vWF (SC‐8068) were purchased from Santa Cruz, USA. KDR (MAB3571), β‐actin (MAB8929), recombinant FSTL3 (AF1288‐F3), and Proteome Profiler Array Human Angiogenesis Array Kit (ARY007) were purchased from R&D, USA.

### Human iPS Cells Differentiation

Four different clones of human iPS Cells were differentiated using StemPro‐34 SFM serum free media (Thermo Fisher Scientific) supplemented with BMP4 (Thermo Fisher Scientific), Activin A (R&D), fibroblast growth factor (FGF) (Miltenyi Biotec, Germany), and vascular endothelial growth factor (VEGF) (Thermo Fisher Scientific) for 5 days. The differentiated cells were seeded on collagen IV (R&D), while CD144 positive cells were magnetically sorted on day 6 using MicroBeads Kit (Miltenyi BIotec) and cultured in EGM‐2 media (LONZA) (iPS‐ECs). *FSTL3* was overexpressed or knocked down by transfection or lentiviral gene transfer in iPS‐ECs and the cells were harvested 2–3 days later for further analysis or used for in vivo angiogenesis and hind limb ischemia assays. Equal cell numbers were used between control and treated conditions in all experiments in this study.

#### In Vivo Matrigel Plug Assay

In in vivo angiogenesis assays, iPS‐ECs overexpressing *FSTL3* (EX‐FSTL3) or control plasmid (EX‐mCherry) were mixed with 50 μl of Matrigel and injected subcutaneously into the back or flank of NOD.CB17‐Prkdcscid/NcrCrl severe combined immunodeficiency (SCID) mice. Six injections were conducted for each group. Seven days later, the mice were sacrificed and the plugs were harvested, frozen in liquid nitrogen, and cryosectioned. Samples were fixed with 4% paraformaldehyde in phosphate buffered saline (PBS) at 4°C overnight, and then H&E staining was performed. Images were assessed with Axioplan 2 imaging microscope with Plan‐NEOFLUAR ×10, NA 0.3, objective lenses, AxioCam camera, and Axiovision software (all Carl Zeiss MicroImaging, Inc., Germany).

### Experimental Hind Limb Ischemia

The mouse hind limb ischemia model was performed as previously described 19, 20. iPS‐ECs overexpressing *FSTL3* (EX‐FSTL3) or control plasmid (EX‐mCherry) were trypsinized and injected intramuscularly into the adductors of ischemic NOD.CB17‐Prkdcscid/NcrCrl SCID mice. Tissue blood flow of both legs was sequentially assessed by Laser Doppler imaging (moorLDL2‐IR).

### Statistical Analysis

Data is expressed as mean ± SEM and analyzed using GraphPad Prism 5 software with a two‐tailed Student's *t* test for two groups or pairwise comparisons or analysis of variance (ANOVA). A value of *, *p* < .05; **, *p* < .01; ***, *p* < .001 was considered significant.

The detailed “Methods and Materials” are given in “Supporting Information Appendix Experimental Procedures” section.

## Results

### iPS Cells Differentiation Toward ECs

**Figure 1:**
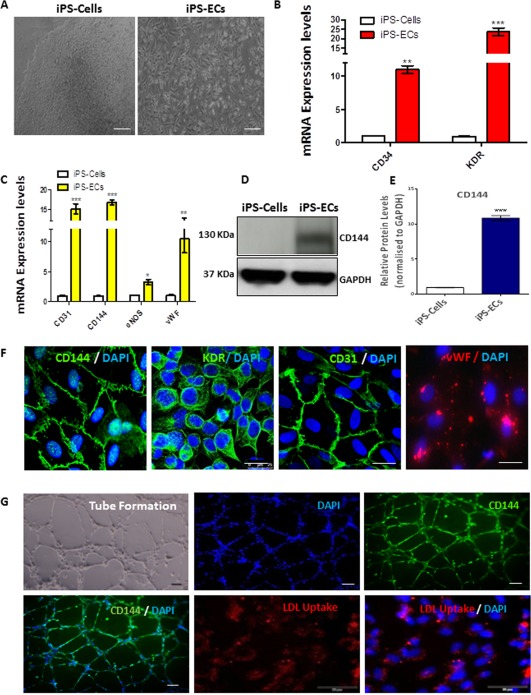
Induced pluripotent stem cell differentiation toward endothelial cells. Human iPS Cells were differentiated using StemPro serum free media supplemented with BMP4, Activin A, fibroblast growth factor (FGF), and vascular endothelial growth factor (VEGF) for 5 days. The differentiated cells were seeded on collagen IV, while CD144 positive cells were magnetically sorted on day 6 using MicroBeads Kit (Miltenyi BIotec) and culturing in EGM‐2 media (LONZA). **(A)**: Images show morphology of iPS Cells (left panel) and of their differentiated EC counterparts (right panel) Scale bar: 50 μm. **(B)**: Real time polymerase chain reaction (PCR) data revealed significantly increased early and **(C)** late EC marker mRNA expression (Data is means ±SEM [*n* = 3], *, *p* < .05; **, *p* < .01; ***, *p* < .001). **(D)**: Western blots showing protein expression of the EC‐specific marker CD144 in iPS‐ECs and **(E)** corresponding densitometry. **(F)**: Immunofluorescent confocal images showing that iPS‐ECs express typical EC markers, CD144, KDR, CD31, and vWF, also costained with DAPI (4′,6‐diamidino‐2‐phenylindole, dihydrochloride). **(G)**: iPS‐ECs stained positive for CD144, were able to uptake LDL and form tubes in vitro. Scale bar: 50 μm. The data presented are representative or means (±SEM) of three independent experiments. Abbreviations: DAPI, 4′,6‐diamidino‐2‐phenylindole; EC, endothelial cell; GAPDH, Glyceraldehyde 3‐phosphate dehydrogenase; iPS, induced pluripotent stem; LDL, low density lipoprotein.

The model of iPS Cells generation and subsequent EC differentiation have been implemented in follow‐up experiments where somatic cells were reprogramed to a pluripotent state according to a standard protocol 21, and as previously described, giving rise to iPS Cells 22, and Supporting Information Figure S1A–S1C. iPS Cells were differentiated to iPS‐ECs by selecting the CD144 positive population, seeding on collagen IV and culturing in EGM‐2 media. iPS‐ECs adopted a distinctive cobble‐stone morphology (Fig. 1A). The potential of the iPS Cells to differentiate toward ECs was confirmed at both mRNA (Fig. 1B, 1C) and protein level (Fig. 1D, and quantification in Fig. 1E), demonstrating that iPS‐ECs exhibited expression of typical early (CD34, KDR) and late EC markers (CD31, CD144, eNOS, and vWF). Immunofluorescent confocal microscope images also revealed that iPS‐ECs expressed characteristic EC markers such as CD144, KDR, CD31, and vWF (Fig. 1F). They also exhibited typical EC tube formation on Matrigel as well as CD144 staining and acetylated‐low density lipoprotein uptake (Fig. 1G). This data confirms the successful generation of iPS‐ECs.

### FSTL3 Is Highly Expressed in iPS‐ECs

**Figure 2:**
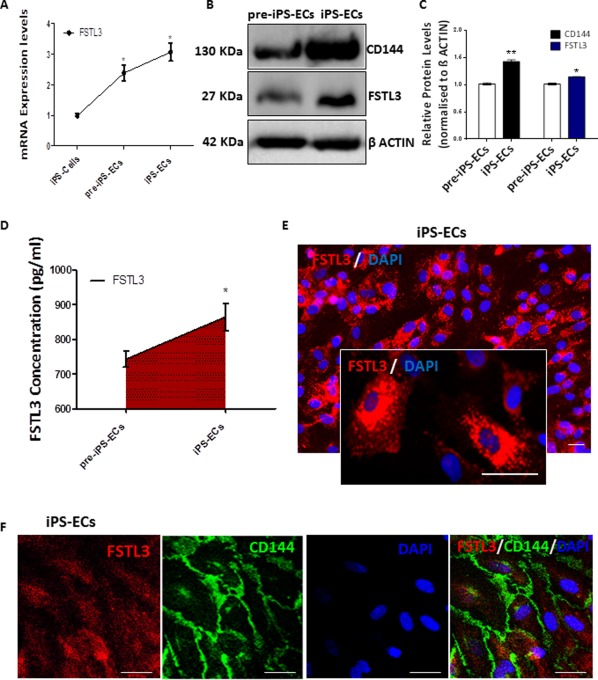
FSTL3 is highly expressed in iPS‐ECs. **(A)**: FSTL3 was progressively induced during EC differentiation in iPS‐ECs at the mRNA level, both preselection (pre‐iPS‐ECs) and postselection with CD144 (iPS‐ECs) (Data is means ± SEM [*n* = 3], *, *p* < .05). **(B)**: Western Blot showing FSTL3 protein being expressed in parallel with the EC‐specific marker CD144 and **(C)** corresponding densitometry. **(D)**: FSTL3 secretion was progressively induced in cell culture media, as shown by ELISA (Data is means ± SEM [*n* = 3], *, *p* < .05). **(E)**: Immunofluorescent images of FSTL3 in iPS‐ECs, Scale bar: 50 µm. **(F)**: Immunofluorescent staining showing that FSTL3 is expressed in parallel with the EC‐specific marker CD144 in iPS‐ECs. Scale bar: 50 µm. The data presented are representative or means (±SEM) of three independent experiments. Abbreviations: DAPI, 4′,6‐diamidino‐2‐phenylindole; ECs, endothelial cells; FSTL3, follistatin‐like 3; iPS, induced pluripotent stem.

To start elucidating the role of FSTL3 in iPS‐ECs, initial real time experiments using equal numbers of cells, revealed that FSTL3 was induced during EC differentiation from iPS Cells both before the selection (at day 6 of EC differentiation) of CD144 positive cells (pre‐iPS‐ECs) and after the selection of CD144 positive cells (iPS‐ECs) (Fig. 2A). These results were also confirmed in protein levels as Western blots data showed (Fig. 2B, and quantification in Fig. 2C) a parallel expression of FSTL3 and CD144. Interestingly, FSTL3 was also extracellularly secreted in both pre‐iPS‐ECs and iPS‐ECs as enzyme‐linked immunosorbent assay (ELISA) assays revealed in Figure 2D when the culture media were assessed. FSTL3 was highly expressed in iPS‐ECs as confirmed by immunofluorescent confocal microscopy (Fig. 2E). Furthermore, immunofluorescent confocal microscopy demonstrated that staining of FSTL3 was concomitant with the EC‐specific marker CD144 (Fig. 2F), indicating that FSTL3 expression may be an important event in iPS‐ECs. Taken together, these results demonstrate that FSTL3 expression is induced during generation of iPS‐ECs and indicate a possible key role of FSTL3 in these cells.

### FSTL3 Regulates EC Marker Expression in iPS‐ECs

**Figure 3:**
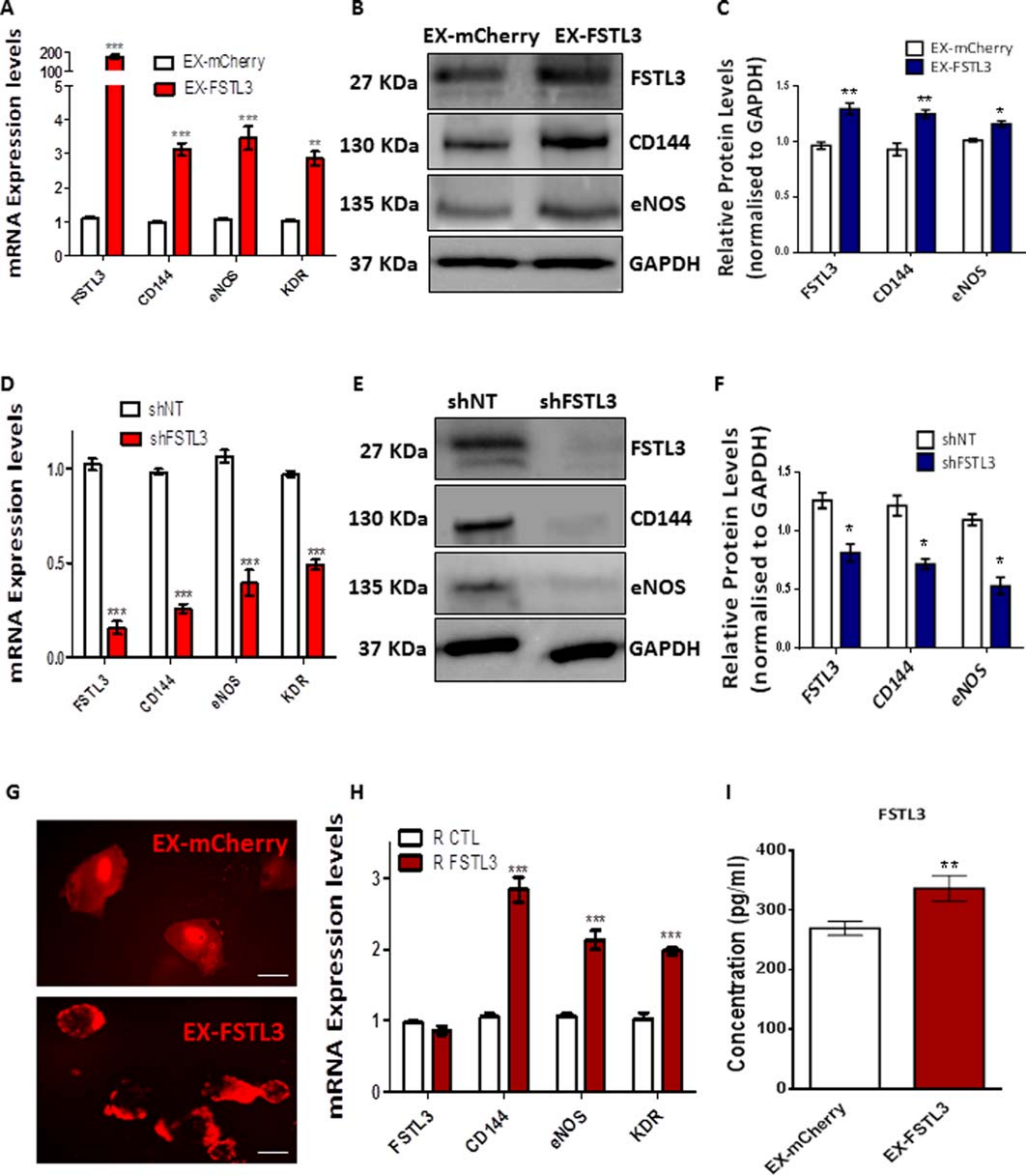
FSTL3 regulates endothelial cell (EC) marker expression in induced pluripotent stem (iPS)‐ECs. iPS‐ECs were transfected with the EX‐FSTL3 plasmid carrying the red mCherry fluorophore, or an empty control vector plasmid (EX‐mCherry). Forty‐eight hours later, *FSTL3* overexpression induced **(A)** mRNA and **(B, C)** protein expression of EC markers CD144, eNOS, and KDR (Data is means ± SEM [*n* = 3], **, *p* < .01; ***, *p* < .001). FSTL3 knockdown by short hairpin RNA (shRNA) in iPS‐ECs resulted in suppression of EC markers at the **(D)** mRNA and **(E, F)** protein levels when the cells were harvested 72 hours later (Data is means ± SEM [*n* = 3], ***, *p* < .001). **(G)**: Fluorescent microscope images of iPS‐ECs transfected with EX‐FSTL3 plasmid carrying the red mCherry fluorophore, or an empty control vector plasmid (EX‐mCherry). Scale bar: 50 µm. **(H)**: Treatment of the cells with 25–50 ng/ml of recombinant FSTL3 (R FSTL3) increased EC marker expression such as CD144, eNOS, and KDR compared to phosphate buffered saline (PBS) control (R CTL) (Data is means ± SEM [*n* = 3], ***, *p* < .001). ELISA showing FSTL3 concentration in cell culture media after *FSTL3* overexpression compared to control (Data is means ± SEM [*n* = 3], **, *p* < .01). The data presented are representative or means (±SEM) of three independent experiments. Abbreviations: FSTL3, follistatin‐like 3; GAPDH, glyceraldehyde 3‐phosphate dehydrogenase. (I) Enzyme‐linked immunosorbent assay (ELISA) showed increased concentration of FSTL3 in the media after overexpression with EX‐FSTL3 plasmid (Data is means ± SEM [n < 3], **, *p* < .01

Since expression of FSTL3 was shown to increase in parallel with EC markers in iPS‐ECs, we sought to further clarify the role of FSTL3 in iPS‐ECs. Consequently, we examined whether manipulating the gene expression of *FSTL3* by overexpression or knockdown experiments or by supplementing the differentiating cells with recombinant FSTL3 protein in the media, would influence the EC marker expression. Initially, in order to assess whether FSTL3 may regulate the EC marker expression during this process, *FSTL3* was overexpressed in iPS‐ECs by transfection or lentiviral gene transfer. Forty eight hours later, the cells were harvested for further analysis. Since the plasmids were tagged with the mCherry fluorescent marker, the transfection/infection efficiency could be visualized by fluorescent microscopy (Fig. 3G). Overexpression of *FSTL3* significantly increased the expression of EC markers CD144, eNOS, and KDR (Fig. 3A, 3B, and quantification in Fig. 3C) at both the mRNA and protein levels, respectively. Importantly, when FSTL3 was silenced by short hairpin RNA (shRNA) in iPS‐ECs, and the cells were harvested 72 hours later, there was a significant downregulation of EC markers in both mRNA and protein levels, respectively (Fig. 3D, 3E, and quantification in Fig. 3F). The involvement of extracellular FSTL3 (R FSTL3) in iPS‐ECs was evidenced by stimulation using recombinant FSTL3, which resulted in a significant increase in expression of the EC markers (Fig. 3H). Higher levels of extracellular FSTL3 was also observed in iPS‐ECs overexpressing *FSTL3*, as shown by ELISA assay (Fig. 3I).

### FSTL3 Expression Is Regulated by KLF4

**Figure 4:**
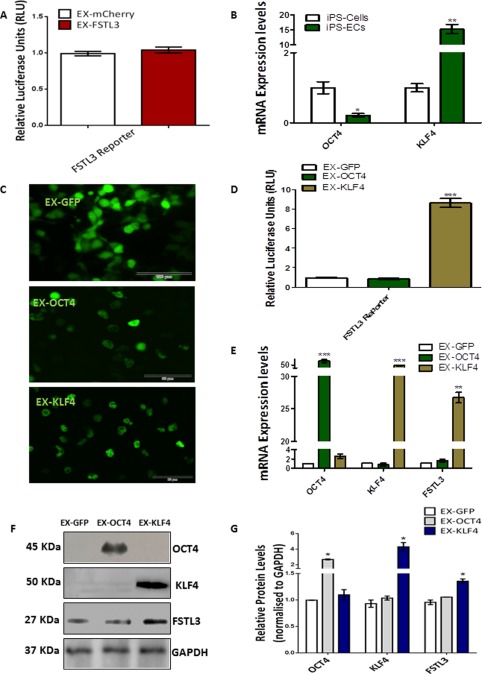
FSTL3 expression is regulated by KLF4. **(A)**: Luciferase assay of the FSTL3 promoter activity showed no significant changes when *FSTL3* was overexpressed compared to empty vector control in iPS‐ECs (Data is means ± SEM [*n* = 3]). **(B)**: During iPS‐ECs generation, KLF4 expression at the mRNA level was increased with a concomitant decrease of the pluripotent marker OCT4 (Data is means ± SEM [*n* = 3], *, *p* < .05; **, *p* < .01). **(C)**: Images of 293‐T cells (Scale bar: 100 μm) 48 hours after transfection with GFP‐tagged plasmids carrying the transcription factors *OCT4* or *KLF4* compared to control (GFP). **(D)**: Luciferase assay in iPS‐ECs showed significant increase in the FSTL3 promoter activity after overexpression with *KLF4*, but not OCT4 (Data is means ± SEM [*n* = 3], ***, *p* < .001). **(E)**: mRNA expression levels 48 hours after transfection with GFP‐tagged plasmids carrying the transcription factors OCT4 or KLF4 compared to control (GFP) (Data is means ± SEM [*n* = 3], **, *p* < .01; ***, *p* < .001). **(F)**: Western blot and **(G)** its corresponding densitometry, showing FSTL3 increasing in the protein level in parallel with overexpression of *KLF4* but not *OCT4*. The data presented are representative or means (±SEM) of three independent experiments. Abbreviations: ECs, endothelial cells; FSTL3, follistatin‐like 3; GAPDH, Glyceraldehyde 3‐phosphate dehydrogenase; GFP, green fluorescent protein; iPS, induced pluripotent stem.

To shed more light on the signaling surrounding FSTL3 activation in iPS‐ECs, the FSTL3 promoter activity was assessed using Luciferase assays. Overexpression of *FSTL3* had no autoregulatory effects on the FSTL3 promoter in iPS‐ECs (Fig. 4A). We 23 and others 24 have previously shown that KLF4 is implicated in EC differentiation from pluripotent stem cells. In our iPS‐ECs system, it was confirmed that KLF4 was increased during EC differentiation, with a concomitant decrease of the pluripotent marker OCT4 (Fig. 4B). The next question was whether FSTL3 may be regulated by KLF4. Therefore, additional experiments were conducted to clarify the mechanisms by which FSTL3 could be induced in iPS‐ECs generation. Specifically, cells were transfected with GFP‐tagged plasmids carrying KLF4 or OCT4 (Fig. 4C), the latter serving as an additional control, and the levels of FSTL3 expression were observed. FSTL3 levels increased with overexpression of *KLF4* at both mRNA (Fig. 4E), and protein levels (Fig. 4F, and quantification in Fig. 4G) indicating that *KLF4* mediates the induction of FSTL3. Further experiments in iPS‐ECs confirmed that the FSTL3 promoter is regulated by the *KLF4* transcription factor, as Luciferase assays revealed (Fig. 4D). A negative control such as *OCT4* was included in these experiments, showing no effect on FSTL3. In conclusion, our data demonstrates that FSTL3 is transcriptionally activated by *KLF4* during generation of iPS‐ECs.

### FSTL3 Regulates EC Marker Expression iPS‐ECs by Facilitating β‐Catenin Nuclear Translocation Through Inhibition of GSK3β Activity

Since FSTL3 was shown to modulate induction of EC markers, including those particularly localized at cell junctions such as CD144, we conducted further experiments to elucidate underlying mechanisms in iPS‐ECs. There is prior evidence implicating β‐catenin as a key mediator of EC function and being a major constituent of cell‐cell junctions 25. In dormant cells, β‐catenin is associated with CD144 mainly at the membrane, maintaining low cytoplasmic levels. When released into the cytoplasm, β‐catenin can either translocate to the nucleus or incorporate into a cytoplasmic complex, where GSK3β is a key component, leading to ubiquitination and proteasomal degradation of β‐catenin 26. If GSK3β is blocked or inactivated, the degradation of β‐catenin is attenuated, favoring its nuclear translocation 26. When in the nucleus, β‐catenin plays a role in the activation of T‐cell factor/lymphoid enhancer factor (LEF) 27 and it also modulates vascular remodeling 28.

**Figure 5:**
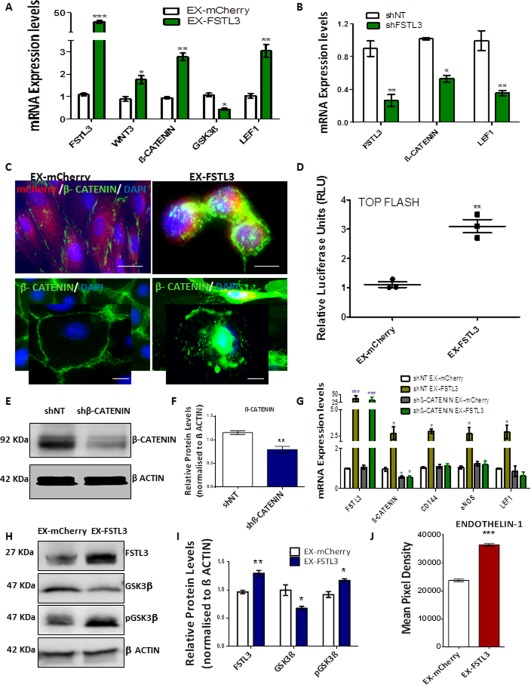
FSTL3 regulates endothelial cell (EC) marker expression in induced pluripotent stem (iPS)‐ECs by facilitating β‐catenin nuclear translocation through inhibition of GSK3β activity. **(A)**: FSTL3 induced expression of Wnt3, β‐catenin, and LEF1 in iPS‐ECs, while suppressing GSK3β expression levels (Data is means ± SEM [*n* = 3], *, *p* < .05; **, *p* < .01; ***, *p* < .001). **(B)**: Knockdown of FSTL3 in iPS‐ECs by short hairpin RNA(shRNA) significantly reduced the expression of β‐catenin and LEF1 (Data is means ± SEM [*n* = 3], *, *p* < .05; **, *p* < .01). **(C)**: Immunofluorescent confocal images showing enhanced β‐catenin translocation to the cell nucleus of *FSTL3*‐overexpressing cells compared to control (EX‐mCherry), Scale bar: 50 μm. **(D)**: Luciferase assay showed increased Top Flash promoter activity for cells overexpressing *FSTL3* compared to control (EX‐mCherry) (Data is means ± SEM [*n* = 3], **, *p* < .01). **(E)**: Western blot and its **(F)** corresponding densitometry, showing β‐catenin knockdown by shRNA. **(G)**: β‐catenin was knocked down by shRNA and *FSTL3* was overexpressed the following day in iPS‐ECs. Real Time polymerase chain reaction (PCR) data reveal the induction of EC markers and LEF1 mediated by FSTL3 was ablated by β‐catenin knockdown (Data is means ± SEM [*n* = 3], *, *p* < .05; ***, *p* < .001). **(H)**: *FSTL3* overexpression in iPS‐ECs led to GSK3β decrease and phospho‐GSK3β (pGSK3β) increase in the protein level, as shown by Western blot and its **(I)** corresponding densitometry. **(J)**: Human angiogenesis Proteome Profiler array analysis revealed the overexpression of *FSTL3* leads to induced expression of the angiogenic factor Endothelin‐1 (Data is means ± SEM [*n* = 3], ***, *p* < .001). The data presented are representative or means (±SEM) of three independent experiments. Abbreviations: FSTL3, follistatin‐like 3; GSK3β, glycogen synthase kinase‐3β; pGSK3β, phospho‐GSK3β.

To determine precisely how FSTL3 was involved in enhancing EC markers, *FSTL3* was overexpressed in iPS‐ECs, and the cells were harvested 48 hours later for further analysis. Overexpression of *FSTL3* led to significant upregulation in expression of WNT3, β‐catenin, and LEF1, and a significant reduction in GSK3β (Fig. 5A). When FSTL3 expression was knocked down by shRNA in iPS‐ECs, as it has been described above, this resulted in significant suppression of β‐catenin and LEF1 (Fig. 5B). Confocal microscopy of iPS‐ECs showed that FSTL3 was able to enhance β‐catenin translocation to the cell nucleus compared to control (Fig. 5C). Further Luciferase assay experiments (Fig. 5D) confirmed the amplified Top Flash promoter activity by FSTL3, also indicating increased presence of β‐catenin protein levels in the nucleus. Importantly, the induction of EC markers and LEF1 mediated by FSTL3 was ablated when β‐catenin was knocked down by shRNA (Fig. 5G). The knockdown of β‐catenin was also confirmed in the protein level in Figure 5E, and quantification in Figure 5F. In addition, when *FSTL3* was overexpressed, GSK3β was decreased in the protein level while phospho‐GSK3β, the deactivated form, was increased (Fig. 5H, and quantification in Fig. 5I), indicating reduced GSK3β activity. The angiogenic activity of FSTL3 in regards to β‐catenin involvement was also examined using a human angiogenesis Proteome Profiler array, which identified Endothelin‐1 being highly induced after *FSTL3* overexpression in iPS‐ECs (Fig. 5J). These results clearly demonstrate that FSTL3 has a critical role in the regulation of EC markers in iPS‐ECs by facilitating β‐catenin nuclear translocation through inhibition of GSK3β activity.

### FSTL3 Induced Vascular Tube Formation In Vivo

**Figure 6:**
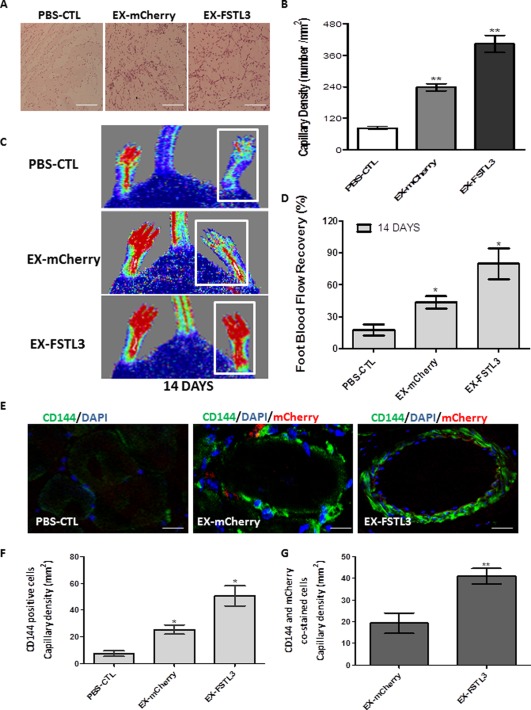
FSTL3 induced vascular tube formation in vivo and significantly improved angiogenesis, neovascularization, and blood flow recovery in the hind limb ischemic model. Human induced pluripotent stem (iPS)‐ECs overexpressing *FSTL3* enhanced the formation of vascular‐like tubes in vivo. **(A)**: iPS‐ECs overexpressing *FSTL3* (EX‐FSTL3), significantly induced angiogenesis, after subcutaneous injection in severe combined immunodeficiency (SCID) mice, in comparison to the control iPS‐ECs (PBS CTL) or control iPS‐ECs expressing *mCherry* (EX‐mCherry), as shown by H&E staining and **(B)** capillary density quantification (Data is means ± SEM [*n* = 3], **, *p* < .01). Scale bar: 50 µm. **(C)**: phosphate buffered saline (PBS)‐CTL and iPS‐ECs overexpressing *mCherry* (EX‐mCherry) or *FSTL3* (EX‐FSTL3) were injected intramuscularly in NOD.CB17‐Prkdcscid/NcrCrl SCID mice immediately after induction of hind limb ischemia. Laser Doppler images show blood flow (BF) in the lower limbs of mice in prone position on day 14. **(D)**: BF recovery in the ischemic foot (calculated as a percentage ratio between ischemic foot BF and the contralateral foot) for each of the conditions. Statistical analysis shows significantly higher BF recovery in the EX‐FSTL3 treated mice at 14 days in comparison to controls; (Data is means ± SEM [*n* = 3], *, *p* < .05). **(E)**: Sections of adductor muscles were stained with CD144 antibody for each condition. **(F)**: Quantified capillary density of CD144‐stained cells, expressed as capillary number per mm^2^ (Data is means ± SEM [*n* = 3], *, *p* < .05). **(G)** Quantified capillary density of CD144‐ and mCherry‐stained cells (Data is means ± SEM; **, *p* < .01; quantification from 10 random microscopic fields at ×40, scale: 50 μm). The data presented are representative or means (±SEM) of three independent experiments. Abbreviations: DAPI, 4′,6‐diamidino‐2‐phenylindole; ECs, endothelial cells; FSTL3, follistatin‐like 3.

To complement the findings from our in vitro data and to confirm in vivo relevance, further experiments were conducted to test whether FSTL3 could induce angiogenesis in vivo. iPS‐ECs were transfected or infected with FSTL3 or control plasmid, and 48 hours later, were mixed with Matrigel and subcutaneously injected in NOD.CB17‐Prkdcscid/NcrCrl SCID mice. Seven days later, the mice were sacrificed, the plugs were harvested and H&E staining was performed. iPS‐ECs overexpressing *FSTL3* enhanced the formation of vascular‐like tubes in vivo in Matrigel plug assays as shown by H&E staining in Figure 6A, and quantification of capillary density number in Figure 6B. This data suggests the notion that FSTL3 improves the function of iPS‐ECs and subsequent angiogenic potential.

### FSTL3 Significantly Improved Neovascularization and Blood Flow Recovery in the Hind Limb Ischemic Model

Further experiments were conducted to test whether FSTL3 could induce angiogenesis in ischemic tissues and improve blood flow recovery. iPS‐ECs overexpressing control *mCherry* (EX‐mCherry) or *FSTL3* (EX‐FSTL3) were intramuscularly injected, along with control PBS (PBS‐CTL), after induction of hind limb ischemia in NOD.CB17‐Prkdcscid/NcrCrl SCID mice. Laser Doppler imaging of blood flow at 14 days postdelivery of iPS‐ECs overexpressing *FSTL3* showed improved blood flow recovery and enhanced neovascularization (Fig. 6C, 6D) in the ischemic limbs compared to the controls. More specifically, engrafted iPS‐ECs overexpressing *FSTL3* displayed a typical and well‐defined vascular architecture when compared to controls, as it is shown by confocal immunofluoresent images for the EC marker CD144 (Fig. 6E). In particular, limbs receiving iPS‐ECs overexpressing *FSTL3* displayed significantly higher capillary numbers, when the cells stained with CD144 were quantified (Fig. 6F). Importantly, an enhanced engraftment ability of the (human exogenously delivered) iPS‐ECs overexpressing *FSTL3* was clearly demonstrated when double positive cells for CD144 and mCherry were quantified (Fig. 6G). There results indicate that iPS‐ECs overexpressing *FSTL3* display characteristic endothelial functions when tested in vivo. In conclusion, our findings demonstrate that FSTL3 has the potential to induce vessel formation in vivo, further supporting its important role in improving the function of iPS‐ECs.

### FSTL3 Induced EC Differentiation and Tube Formation in Pre‐iPS‐ECs Before CD144‐Selection

During the early stages of iPS‐ECs generation (0–6 days before CD144 selection), cells overexpressing *FSTL3* (EX‐FSTL3) induced EC marker expression in pre‐iPS‐ECs compared to control (Ex‐mCherry) (Supporting Information Fig. S2A). Furthermore, when equal number of pre‐iPS‐ECs (40,000 per condition) were seeded on Matrigel (Supporting Information Fig. S2B) for 8 hours, tube formation was evaluated and quantified in control (EX‐mCherry) versus pre‐iPS‐ECs overexpressing *FSTL3* (EX‐FSTL3) using Image J. The total master segments length, total segments, total meshes area, and capillary tube branch points (Supporting Information Fig. S2C–S2F) showed increased tube formation in the pre‐iPS‐ECS overexpressing *FSTL3* (Supporting Information Fig. S2C–S2F). These results demonstrate that FSTL3 has the potential to induce differentiation toward ECs from human iPS cells.

## Discussion

Cell reprograming is a highly promising regenerative strategy to develop novel therapies to improve vascular function in patients with ischemic diseases. The present study has elucidated, for the first time, the remarkable role of FSTL3 in improving the function and neovascularization potential in iPS‐ECs both in vitro and in vivo. Differentiated ECs typically demonstrate limited proliferative capacity, as well as increased instability and senescence 29, which as a result, substantially compromises their clinical application for treatment of vascular disease. Understanding key factors to facilitate maintenance of EC generation is important as we move toward cell therapy, and we have now shown that FSTL3 plays an important role in this process.

Follistatins play diverse roles in cell proliferation, wound healing, and inflammation, and this is also true for FSTL3, a secreted glycoprotein usually associated with cell signaling and transcription 30, which is important in metabolic events such as glucose and lipid homeostasis 31 and is also a key player in regulating hematopoiesis 32. In addition, FSTL3 is an established inhibitor of TGF‐β family members such as Activin A and bone morphogenetic proteins 33.

Given the diverse roles of follistatins regarding function and tissue distribution 10, we sought to determine the role of FSTL3 in iPS‐ECs. In particular, we have investigated the mechanism by which FSTL3 acts to promote enhancement of EC markers and function. This particular candidate was chosen as the only follistatin family member significantly highly expressed in iPS‐ECs, which was also the highest expressing gene compared to other follistatins. Indeed, the findings of the present study demonstrate an important role of FSTL3 in iPS‐ECs, and in the early stages of EC differentiation, which may be significant toward our aim of generating therapeutically relevant ECs for use in both regenerative medicine and treatment of ischemic diseases.

In this study, using the model of iPS‐ECs generation, FSTL3 expression was found to be upregulated by *KLF4*, a key mediator of EC differentiation 23, 53. In addition, FSTL3 expression progressively increased throughout the iPS‐ECs generation process, highlighting a likely angiogenic role. Taken together, this increase in FSTL3 expression by *KLF4* is likely made possible through the physical interaction of *KLF4* with ElK‐1 34; with the latter interacting, in turn, with the FSTL3 promoter, as identified by putative transcription factor binding site research in the TRANSFAC database 35, 36.

Furthermore, we found that FSTL3 serves an important regulatory function in iPS‐ECs, by increasing expression of EC markers, a decrease of which is characteristic of EC dysfunction 37. This increase in FSTL3 also highlights how important FSTL3 may be in enhancing iPS‐ECs generation and maintaining a more stable EC phenotype. Notably, through further experiments, we found that the mechanism by which FSTL3 induced the EC marker expression in our system was through inhibition of GSK3β activity and, therefore, β‐catenin translocation to the nucleus. Indeed, in agreement with these findings, previous studies have shown that increased EC marker expression can be linked to suppression of GSK3β by increased phosphorylation 38, an event followed by β‐catenin translocation from the cytoplasm to the cell nucleus 39. Seeking to further elucidate the mechanism behind the increased GSK3β phosphorylation after *FSTL3* overexpression in our study, angiogenic profiler arrays were performed, which showcased Endothelin‐1 as the most highly expressed angiogenic factor. Endothelin‐1 is a peptide known to induce angiogenic responses in ECs 40 and, most importantly, to increase phosphorylation of GSK3β, resulting in a substantial inhibition of GSK3β activity 41, 42, thus, further validating our observations. Furthermore, β‐catenin is a coactivator of the Wnt family of secreted glycolipoproteins 43 and plays a multifunctional role in key processes, such as cell cycle regulation 44, cell to cell adhesion 45, and gene transcription 46, 47, 48. However, β‐catenin signaling is also widely documented as imperative in the proliferation and survival of ECs as well as in promoting angiogenesis 49 during both development and ischemic disease 50, 51, thereby promoting an increase in EC markers in iPS‐ECs, a fact which is in agreement with our findings.

Remarkably, when tested in two different in vivo models of angiogenesis and hind limb ischemia, iPS‐ECs overexpressing *FSTL3* were able to induce increased angiogenesis. This was indicated by enhanced capillary formation, greatly improved blood flow recovery as well as by increases in the EC‐specific marker (CD144) expression, indicating a key role of FSTL3 in promoting angiogenesis and neovascularization. These results demonstrate the importance of FSTL3‐expressing iPS‐ECs used in in vivo models, exhibiting increased functionality potential. In further agreement with this study, previous research focusing on other follistatins and FSTL proteins has provided supporting evidence that they may play a direct or indirect role in regulating EC function and increasing angiogenesis. For example, follistatin has been reported to promote angiogenesis in the rabbit cornea 17 and also be upregulated in human umbilical vein endothelial cells in response to VEGF, a central migratory, sprouting, and proliferation growth factor 52.

## Conclusion

**Figure 7:**
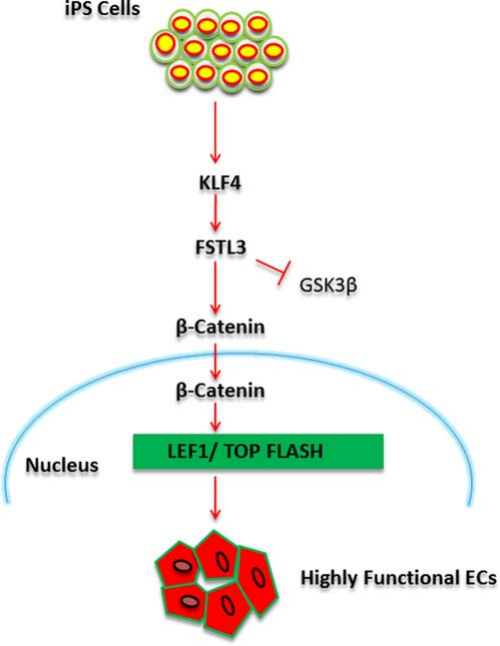
Schematic diagram of FSTL3 enhancing iPS‐EC function facilitating β‐catenin nuclear translocation through inhibition of GSK3β activity. FSTL3 is activated by KLF4 during iPS‐ECs generation and holds a pivotal role in enhancing EC marker expression by enabling β‐catenin nuclear translocation through inhibition of GSK3β activity. This in turn, exerts its own regulatory signaling effects on enhancing EC function. Abbreviations: EC, endothelial cell; FSTL3, follistatin‐like 3; GSK3β, glycogen synthase kinase‐3β; iPS, induced pluripotent stem.

Our data clearly demonstrates that FSTL3, which is regulated by *KLF4*, plays an important role in enhancing EC function of iPS‐ECs. Our observations suggest that FSTL3 facilitates β‐catenin nuclear translocation by inhibition of GSK3β activity through Endothelin‐1, enhancing, in turn, β‐catenin's signaling effects on increasing EC marker expression. The schematic diagram of this proposed mechanism is shown in Figure 7. This novel strategy for improving EC generation based on defined mechanisms could therefore shed further light on the process. In addition, it can have an enormous impact on efficiency and production of therapeutically relevant numbers of cells. The generated ECs could eventually be used for patient‐specific therapy, particularly toward treatment of vascular diseases where EC dysfunction plays a central role.

## Author Contributions

S.K.: conception and design, collection and/or assembly of data, data analysis and interpretation, manuscript writing; M.V.‐G.: collection and/or assembly of data, data analysis and interpretation, manuscript writing; R.C.: Collection and/or assembly of data, Data analysis and interpretation; D.C., M.E., M.T., C.M., A.C., K.O., and C.Y.: collection and/or assembly of data; A.W.S., D.J.G., and L.Z.: provision of study material, final approval of manuscript; A.M.: conception and design, collection and/or assembly of data, data analysis and interpretation, manuscript writing, financial support, final approval of manuscript.

## Disclosure of Potential Conflicts of Interest

The authors indicated no potential conflicts of interest.

## Supporting information

Supplementary Figures

Supplementary Figure Legends S1 AM

Supplementary Figure Legends S1 Marked AM

Experimental Procedures

Supplementary Combined File
