# Supplementary figures and images for: Follistatin‐Like 3 Enhances the Function of Endothelial Cells Derived from Pluripotent Stem Cells by Facilitating β‐Catenin Nuclear Translocation Through Inhibition of Glycogen Synthase Kinase‐3β Activity
Source: Stem Cells. 2018 Apr 10;36(7):1033–44. doi: 10.1002/stem.2820 (PMC6099345; doi:10.1002/stem.2820)

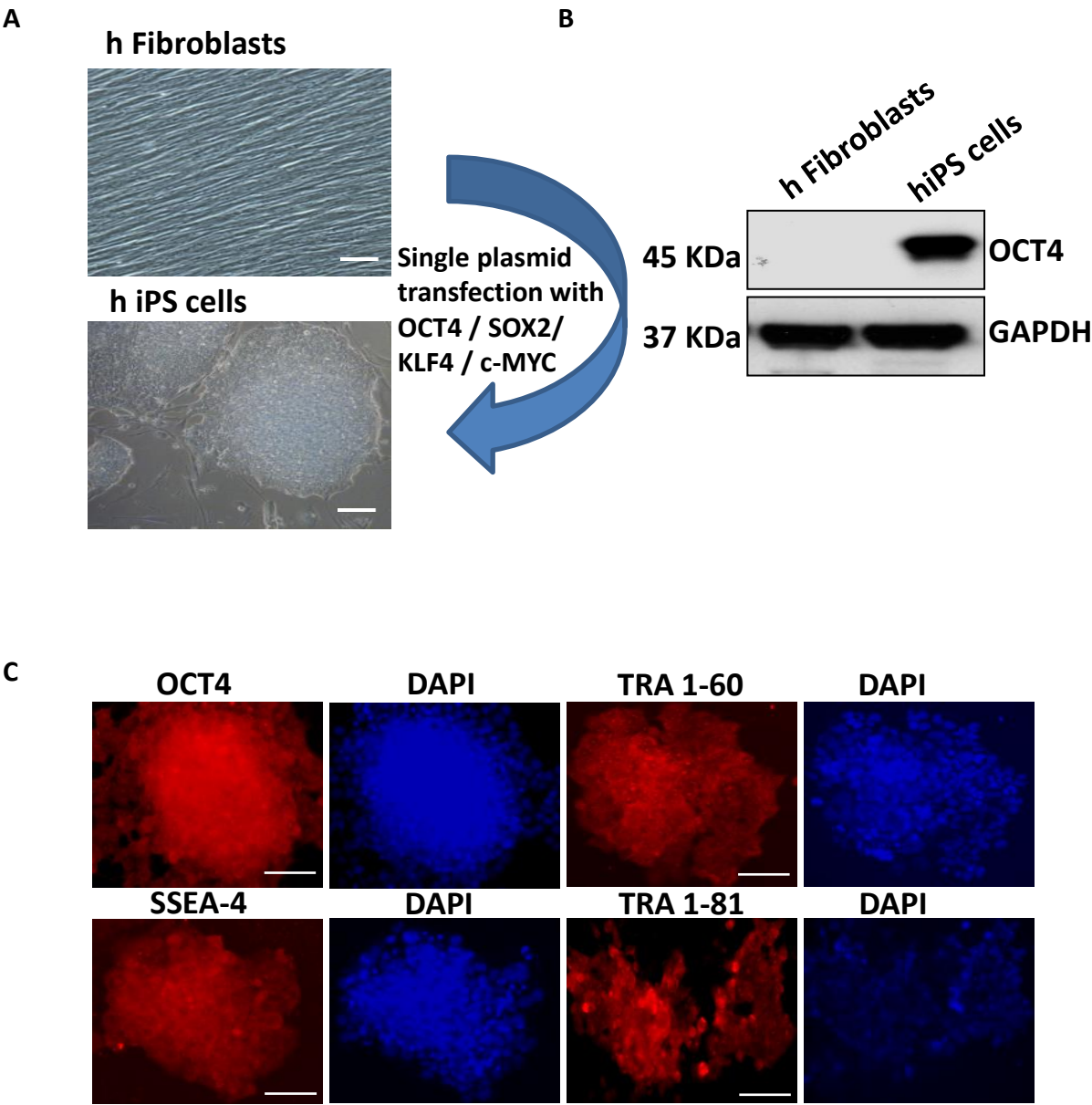

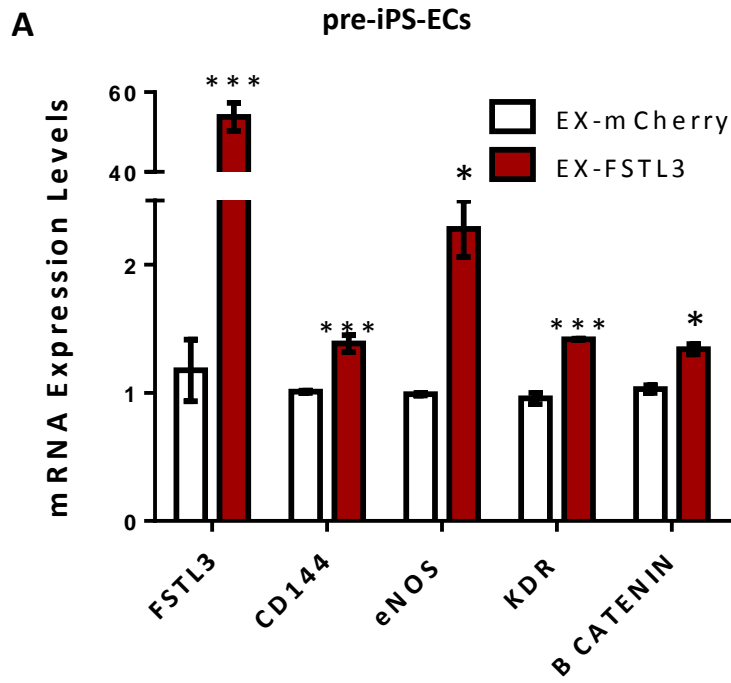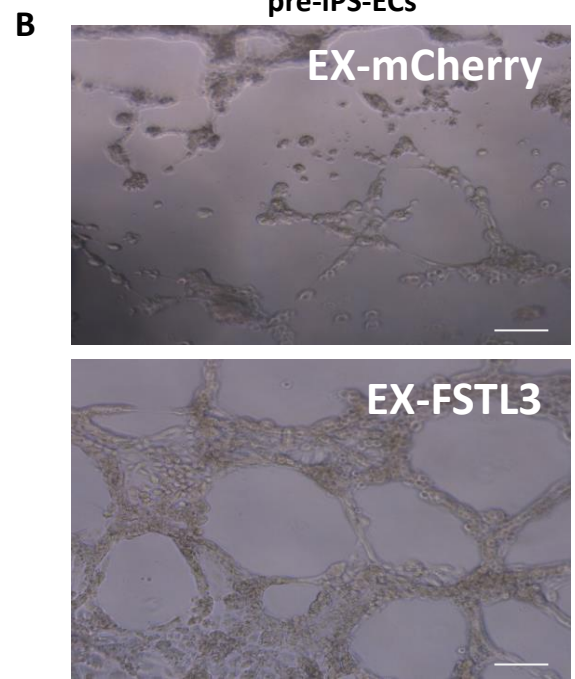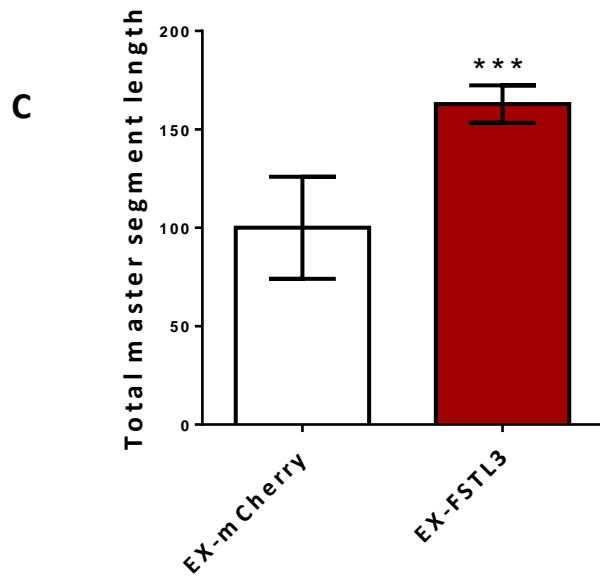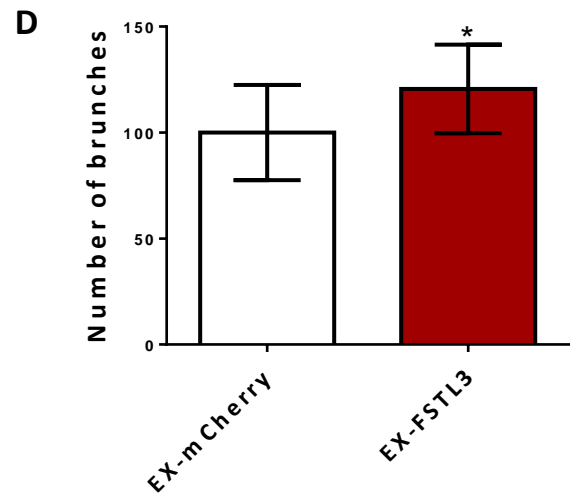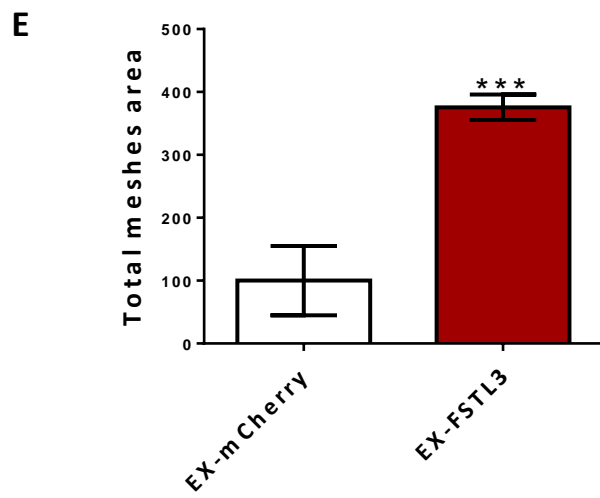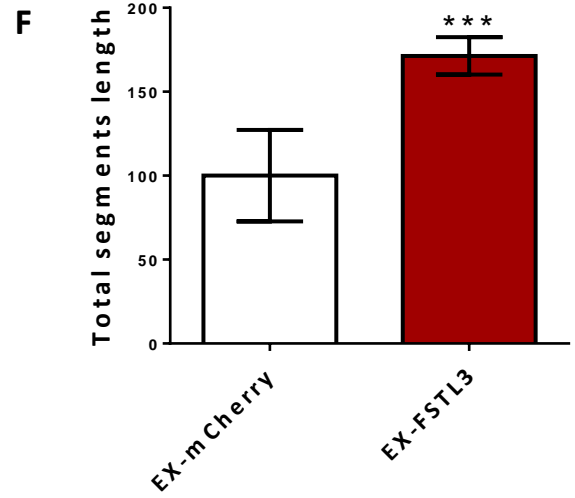

Supplement: Supplementary file 1 — Supplementary Figures [file STEM-36-1033-s001.pdf]
