# Supplementary material for: Follistatin‐Like 3 Enhances the Function of Endothelial Cells Derived from Pluripotent Stem Cells by Facilitating β‐Catenin Nuclear Translocation Through Inhibition of Glycogen Synthase Kinase‐3β Activity
Source: Stem Cells. 2018 Apr 10;36(7):1033–44. doi: 10.1002/stem.2820 (PMC6099345; doi:10.1002/stem.2820)
Supplement: Supplementary file 2 — Supplementary Figure Legends S1 AM [file STEM-36-1033-s002.docx]

***SI Appendix,* Figure Legends**

***Kelaini, at al.***

**Supplementary Figure S1**

**Human iPS cells generation:** iPS cells generated from human fibroblasts using a DNA-free integration method based on a single plasmid transfection of the four reprogramming transcription factors (OCT4, SOX2, KLF4, and c-MYC). (A) Images show fibroblasts and colonies of iPS cells. (B) iPS cells express the pluripotent marker OCT4 in the protein level. (C) Immunofluorescence staining showed a typical staining for pluripotent markers OCT4, TRA1-60, SSEA-4, TRA1-81. 4',6-diamidino-2-phenylindole DAPI was used and stained the cell nucleus. Scale bar, 25 µm.

**Supplementary Figure S2**

**FSTL3 induced** endothelial cell differentiation and tube formation in pre-iPS-ECs before CD144-selection: (A) pre-iPS-ECs overexpressing FSTL3 for 48 hours showed increasing EC markers at the mRNA level (Data is means ±SEM (n=3), **P* < 0.05, ****P* < 0.001). (B) Representative tube formation images of pre-iPS-ECs, previously transfected with either control plasmid (EX-mCherry) of FSTL3 (EX-FSTL3), cultured for 8 hours on Matrigel (Scale bar = 100 µm). Quantification of (C) total master segments length, (D) number of branches, (E) total meshes area, and (F) total segments length. Standard Matrigel was allowed to polymerize in 6-well plates and equal number of cells (40,000 per condition) were seeded. After 8 hours, tube formation was evaluated and photographed in a Leica DMi1 inverted microscope (Leica Microsystems GmbH) using 10× magnification. Quantification of angiogenesis progression was accomplished using the angiogenesis analyzer in Image J by counting the total master segments length, total segments, total meshes area and capillary tube branch points that formed after 8 hours. (Data is means ±SEM (n=3), **P* < 0.05, ****P* < 0.001). The data presented are representative or means (±SEM) of three independent experiments.
