# Supplementary material for: Follistatin‐Like 3 Enhances the Function of Endothelial Cells Derived from Pluripotent Stem Cells by Facilitating β‐Catenin Nuclear Translocation Through Inhibition of Glycogen Synthase Kinase‐3β Activity
Source: Stem Cells. 2018 Apr 10;36(7):1033–44. doi: 10.1002/stem.2820 (PMC6099345; doi:10.1002/stem.2820)
Supplement: Supplementary file 4 — Experimental Procedures [file STEM-36-1033-s004.pdf]

***SI Appendix*, Experimental Procedures**

Kelaini, at al.

Materials

Cell culture media, serum, and cell culture supplements were purchased from ATCC, MerckMillipore, LONZA, and Thermo Fisher Scientific. Antibodies against VE-cadherin (CD144) (ab33168 & STJ96234), VEGFR (ab9530), GAPDH (ab8245), OCT4 (ab19857 & STJ72238), KLF4 (ab72543) eNOS (ab76198), FSTL3 (STJ112317 & AF1288-SP) were purchased from Abcam, St John's Laboratory or R&D. Antibodies against vWF (SC-8068) were purchased from Santa Cruz. KDR (MAB3571), β-actin (MAB8929), recombinant FSTL3 (AF1288-F3) and Proteome Profiler Array Human Angiogenesis Array Kit (ARY007) were purchased from R&D. TRA-1-60 (Cat.No.09-0010), TRA-1-81 (Cat.No.09-0011), and SSEA-4 (Cat.No.09-0006) antibodies were purchased from Stemgent. The secondary antibodies for immunostaining anti-mouse Alexa 568, and anti-rabbit Alexa 488, anti-rabbit Alexa 568, anti-goat Alexa 568, anti-goat alexa 488 were purchased from Thermo Fisher Scientific. The secondary antibodies for Western blotting were purchased from Abcam and Cell Signalling. Recombinant human VEGF was purchased from Thermo Fisher Scientific.

Methods

Human iPS cells generation

Human iPS were generated as we have previously described[1] . Briefly, iPS cells generated from human fibroblasts using a DNA-free integration method based on a single plasmid transfection of the four reprogramming transcription factors (OCT4, SOX2, KLF4, and c-MYC) and fully characterised.

Human iPS Cells differentiation

Four different clones of human iPS Cells were differentiated using StemPro-34 SFM serum free media (Thermo Fisher Scientific) supplemented with BMP4 (Thermo Fisher Scientific), Activin A (R&D), FGF (Miltenyi Biotec) and VEGF (Thermo Fisher Scientific) for 5 days. The differentiated cells were seeded on collagen IV (R&D), whilst CD144 positive cells were magnetically sorted on day 6 using MicroBeads Kit (Miltenyi BIotec) and cultured in EGM-2 media (LONZA) (iPS-ECs). FSTL3 was overexpressed or knocked down by transfection or lentiviral gene transfer in iPS-ECs and the cells were harvested 2-3 days later for further analysis or used for angiogenesis and *in vivo* hindlimb ischemia assays. Equal cell numbers were used between control and treated conditions in all experiments in this study.

Enzyme-linked Immunosorbent Assay

The concentration of FSTL3 released into the supernatant of iPS-ECs or iPS-ECs overexpressing FSTL3 was detected by FSTL3 ELISA kit (Thermo Fisher Scientific; EHFSTL3) according to the manufacturer's procedure.

Plasmid Generation

EX-FSTL3, EX-mCherry control (pReceiver-Lv224), and the FSTL3 human Promoter reporter (217HPRM16010-PG02) were designed and purchased from Genecopoeia. Plasmids containing the FSTL3 gene (NM_005860.2) and its promoter were amplified by incubating in Competent Cells (Promega #JM109) for 30 minutes on ice. After heat shock at 42°C lasting 90 seconds, samples were centrifuged for 2 minutes at 4000 rpm and 200 μl of the supernatant was used to resuspend pellets and spread onto agar plates supplemented with

ampicillin antibiotic. The Plasmids were amplified with mini Plasmid purification kit from QIAGEN.

Transfection

iPS-ECs were transfected with FSTL3 plasmid or empty vectors (tagged with mCherry) using Endofectin Max (Genecopoeia) according to manufacturer's protocol, and cultured in EGM-2 media. The cells were harvested and endothelial marker expression was tested at both transcriptional and protein level. The efficiency of the transfection was around 70% based on fluorescent microscopy visualisation of the mCherry labelled cells and quantification.

Reverse transcriptase-polymerase chain reaction (RT-PCR) and Quantitative RT-PCR

RT-PCR and real time PCR were performed as described previously[2]. Total RNA was extracted using the RNeasy Mini Kit (Qiagen) according to the manufacturer's protocol. 2 µg RNA were reversely transcribed into cDNA with random primer by reverse transcriptase (RT) (Thermo Fisher Scientific). 20-50ng cDNA (relative to RNA amount) was amplified by quantitative RT-PCR. Relative gene expression was determined by quantitative real time PCR, using 2 ng of cDNA (relative to RNA amount) for each sample with the SYBR Green Master Mix in a 10 µl reaction. Ct values were measured using a LightCycler 480 sequence detector (Roche). GAPDH served as the endogenous control to normalize the amounts of RNA in each sample. For each sample, PCR was performed in duplicate in a 384-well reaction plate (LightCycler 480 Roche real time PCR plates). The gene was considered undetectable beyond 35 cycles. The primer sets designed and used in this study are as follows: ***FSTL3****, 5'-CTT CGT GAG CTC CAT GGG-3' and reverse 5'-CAG GCG GTG TCA ATG TTG-3';* ***VE-CADHERIN (CD144)****, forward, 5'-AAGAAACCGCTGATCGGCA-3' and reverse, 5'-*

*TCGGAAGAATTGGCCTCTGTC-3'; **KDR,** 5'-ATAGAAGGTGCCCAGGAAAAG-3' and reverse, 5'-GTCTTCAGTTCCCCTCCATTG-3' 5'-TTTGAAGGTGGAGAGT GCCAG-3'; **GAPDH**, 5'-TGTGATGGGTGTGAACCACGAGAA-3' and reverse, 5'-GAGCCCTTCCACAATG CCAAAGTT-3'; **eNOS**, 5'-TGATGGC GAAGCGAGTGAA-3' and reverse, 5'-ACTCATCCATACACAGGACCCG-3'; **β-CATENIN**, forward, 5'-GTTCAGTTGCTTGTTCGTGC-3' and reverse, 5'-GTTGTGAACATCCCGAGCTAG-3'; **GSK3β**, forward, 5'-GGTCTATCTTAATCTGGTGCTGG-3' and reverse, 5'-TGGATATAGGCTAAACTTCGGAAC-3'; **LEF1**, forward, 5'-GTCAACTCCAAACAAGGCATG-3' and reverse, 5'-CGTGATGGGATATACAGGCTG-3'. **WNT3**, forward: GTGTTAGTGTCCAGGGAGTTC and reverse CATTTGAGGTGCATGTGGTC; **PECAM1 (CD31)**, forward TCAGAAGGACAAGGCGATTG and reverse GTTATGTTGACCACGATGCTG; **CD34**, Forward: AGAAAGGCTGGGCGAAGAC and reverse: TAGCACGTGGTCAGATGCAG; **vWF**, forward ATGAGTATGAGTGTGCCTGC and reverse GTAGATGGTGCTTCGGTGG.*

Immunofluorescence staining

The procedure used for immunofluorescent staining was similar to that described previously[2]. Briefly, cells were fixed with 4% paraformaldehyde or cold methanol and permeabilised with 0.1% Triton X-100 in PBS for 10 minutes and blocked in 5% goat or donkey serum in PBS for 30 minutes at 37˚C. The cells were incubated with primary antibodies for 1 hour at 37˚C. The bound primary antibody was revealed by incubation with the secondary antibody; anti-mouse Alexa 488, and anti-rabbit Alexa 488, anti-rabbit Alexa 568, anti-goat Alexa 568, at 37˚C for 45 min. Cells were counterstained with 4',6-diamidino-2-phenylindole (DAPI; Sigma-Aldrich), mounted in Vectashield (Vector Laboratories, Inc. USA), and examined with a fluorescence microscope (Axioplan 2 imaging; Zeiss) or SP5 confocal microscope (Leica, Germany).

Immunoblotting

The method used was similar to that described previously[2]. Cells were harvested and washed with cold PBS, re-suspended in lysis buffer (25mM Tris-Cl pH 7.5, 120mM NaCl, 1 mM EDTA pH 8.0, 0.5% Triton X100) supplemented with protease inhibitors (Roche) and lysed by ultra-sonication (twice, 6 seconds each) (Bradson Sonifier150) to obtain whole cell lysate. The protein concentration was determined using the Biorad Protein Assay Reagent. 50 µg of whole lysate was applied to SDS-PAGE and transferred to Hybond PVDF membrane (GE Health), followed by standard western blot procedure. The bound primary antibodies were detected by the use of horseradish peroxidase (HRP)-conjugated secondary antibody and the ECL detection system (GE Health). The densitometry analysis of the bands in the western blots was done using the software Image J by National Institutes of Health (NIH).

Luciferase Reporter Assay

For the luciferase reporter assays, iPS-ECs were seeded into 12-well plates and co-transfected with EX-FSTL3, and control plasmids with the FSTL3 human promoter reporter (217HPRM16010-PG02) (Genecopoeia), or TopFlash promoter[2]. Briefly, 0.33 µg/well of the reporter plasmids were co-transfected with EX-FSTL3, EX-OCT4, EX-KLF4[3] and controls; EX-GFP or EX-mCherry (0.17 µg/well) using Fugene 6 or Endofectin Max according to the manufacturer's protocol. pGL3-Luc Renilla (0.1µg/well) was included in all transfection assays as an internal control. Luciferase and Renilla (Promega) activity assays were detected 48 hours after transfection using a standard protocol[3]. Relative luciferase units (RLU) were defined as the ratio of luciferase activity to Renilla activity with that of control set as 1.0.

FSTL3 treatment

iPS-ECs were treated by addition of human recombinant FSTL3 (25-50 ng/ml) in the cell culture media and the cells were harvested 48 hours later and subjected to further analysis.

Angiogenesis Array

Cell culture supernatants were used for these arrays. iPS-ECs were transfected with either EX-mCherry, or EX-FSTL3 using Endofectin Max as described before. Forty eight hours after transfection, the media were collected and spun at 1000 rpm. The supernatants were collected and either used straight away or frozen in aliquots. Three repeats of each condition were pulled together in each membrane for the arrays. The angiogenesis arrays were performed using the Proteome Profiler Human Angiogenesis Array Kit (ARY007, Bio-Techne Ltd.) according to the manufacturer's instructions. The blots were imaged using SYNGENE G:BOX XX6 and analyzed with HLImage++.

Endothelial cell tube formation

The angiogenic effect of FSTL3 *in vitro* was visualized by analyzing *in vitro* increase in tube formation. Previously transfected with either control plasmid (EX-mCherry) of FSTL3 (EX-FSTL3), pre-iPS-ECs were cultured for 8 hours on polymerized Matrigel at 37 °C. Standard Matrigel was allowed to polymerize in 6-well plates and equal number of cells (40,000 per condition) were seeded in DM. After 8 hours, tube formation was evaluated and photographed in a Leica DMi1 inverted microscope (Leica Microsystems GmbH) using 10× magnification. Quantification of angiogenesis progression was accomplished using the angiogenesis analyzer in Image J by counting the total master segments length, total segments, total meshes area and capillary tube branch points that formed after 8 hours.

Lentiviral particle transduction

Lentiviral particles were produced using MISSION shFSTL3 plasmid DNA (Sigma-Aldrich) according to the protocol provided and as previously described[2]. The shRNA Non-Targeting (shNT) vector was used as a negative control. Briefly, 293-T cells were transfected with the lentiviral vector and the packaging plasmids, pCMV-dR8.2 and pCMV-VSV-G (both obtained from Addgene) using Fugene 6 or Endofectin Lenti. The supernatant containing the lentivirus was harvested 48 hours later, filtered, aliquoted and stored at -80°C. p24 antigen ELISA (Zeptometrix) was used to determine the viral titre. The Transducing Unit (TU) was calculated using the conversion factor recommended by the manufacturer (10^4 physical particles per pg of p24 and 1 transducing unit per 10^3 physical particles for a VSV-G pseudotyped lentiviral vector), with 1 pg of p24 antigen converted to 10 Transducing Units (TU). For the lentiviral particle transduction for the constructs purchased from Genecopoeia, a similar approach was used following the manufacturer's instructions. For lentiviral infection, iPS-ECs were incubated with shFSTL3 or shNT control ($1x10^7$TU/ml) in complete medium supplemented with 10 µg/ml of Polybrene for 24 hours. Fresh medium was added to the cells and the plates were harvested 48 hours later for further analysis. The efficiency of the infection was 70-80%.

LDL Uptake

To detect acetylated low-density lipoprotein (LDL) uptake by iPS-ECs, cells were incubated with Dil-ac-LDL (Molecular Probes) for 4 hours and were examined and photographed under a fluorescent microscope.

***In vitro* tube formation assay**

24-well plates were coated with 289 µl/ml of Matrigel Matrix (10 mg/ml). The plates were incubated at 37ºC for 30 minutes, after which the remaining liquid was removed. $1.2x10^5$ cells were plated in each well at a concentration of $4\ x10^5$ cells/ml. The cells were incubated for up to 18 hours at 37ºC & 5% CO_2. Staining of the tubes was performed as described in the immunofluorescence staining section.

In vivo Matrigel Plug assay

In *in vivo* angiogenesis assays iPS-ECs overexpressing FSTL3 (EX-FSTL3) or control plasmid (EX-mCherry) were mixed with 50 µl of Matrigel and injected subcutaneously into the back or flank of NOD.CB17-Prkdcscid/NcrCrl mice. Six injections were conducted for each group. Seven days later, the mice were sacrificed and the plugs were harvested, frozen in liquid nitrogen, and cryosectioned. Samples were fixed with 4% paraformaldehyde in PBS at 4°C overnight, and then Heamatoxylin & Eosin (H&E) staining was performed. Images were assessed with Axioplan 2 imaging microscope with Plan-NEOFLUAR 10×, NA 0.3, objective lenses, AxioCam camera, and Axiovision software (all Carl Zeiss MicroImaging, Inc.). All procedures were performed in accordance with the Guidance on the Operation of the Animals (Scientific Procedures) Act, 1986 (UK) and were approved by the Queen's University Belfast Animal Welfare and Ethical Review Body.

Experimental hindlimb ischemia

The mouse hindlimb ischemia model was performed as previously described[3, 4]. iPS-ECs overexpressing FSTL3 (EX-FSTL3) or control plasmid (EX-mCherry) trypsinised and injected

intramuscularly into the adductors of ischemic NOD.CB17-Prkdcscid/NcrCrl mice. PBS-CTL was used as an additional control. Tissue blood flow of both legs was sequentially assessed by Laser Doppler imaging (moorLDL2-IR). Fourteen days later, mice were sacrificed, and hindlimb muscles were harvested following in situ perfusion fixation at physiological pressure, frozen in liquid nitrogen, and cryo-sectioned for assessment of neoangiogenesis. Sections of adductor muscles were stained with CD144 antibody and capillary density was expressed as capillary number per mm^2. In brief, specimens were placed in a humidified chamber and blocked in 5% donkey serum in PBS for 30 minutes at 37˚C and incubated with primary antibodies rabbit anti-CD144, prior to immunostaining, as described above. The bound primary antibodies were revealed by incubation with the secondary antibody; anti-rabbit Alexa488, at 37°C for 45 minutes. Specimens were counterstained with 4',6-diamidino-2-phenylindole (DAPI; Sigma-Aldrich), mounted in Floromount-G (Cytomation; DAKO, Glostrup, Denmark), and examined with a fluorescence microscope (Axioplan 2 imaging; Zeiss) or SP5 confocal microscope (Leica, Germany). Immunostaining was assessed and capillary density was calculated as capillary number/mm^2. Cell engraftment ability was assessed by counting cells double positive for mCherry and EC marker (CD144) at ten randomly selected microscopic fields (at x40).

Statistical Analysis

Data is expressed as mean±SEM and analyzed using GraphPad Prism 5 software with a two-tailed Student's t test for two groups or pairwise comparisons or ANOVA. A value of *p<0.05, **p<0.01, ***p<0.001 was considered significant.

References

1. Cochrane A, Kelaini S, Tsifaki M, Bojdo J, Vila-Gonzalez M, Drehmer D, Caines R, Magee C, Eleftheriadou M, Hu Y, Grieve D, Stitt AW, Zeng L, Xu Q, Margariti A. Quaking is a key regulator of endothelial cell differentiation, neovascularization, and angiogenesis. *Stem cells*. 2017;35:952-966
2. Margariti A, Zampetaki A, Xiao Q, Zhou B, Karamariti E, Martin D, Yin X, Mayr M, Li H, Zhang Z, De Falco E, Hu Y, Cockerill G, Xu Q, Zeng L. Histone deacetylase 7 controls endothelial cell growth through modulation of beta-catenin. *Circ Res*. 2010;106:1202-1211
3. Margariti A, Winkler B, Karamariti E, Zampetaki A, Tsai TN, Baban D, Ragoussis J, Huang Y, Han JD, Zeng L, Hu Y, Xu Q. Direct reprogramming of fibroblasts into endothelial cells capable of angiogenesis and reendothelialization in tissue-engineered vessels. *Proc Natl Acad Sci U S A*. 2012;109:13793-13798
4. Emanueli C, Monopoli A, Kraenkel N, Meloni M, Gadau S, Campesi I, Ongini E, Madeddu P. Nitropravastatin stimulates reparative neovascularisation and improves recovery from limb ischaemia in type-1 diabetic mice. *Br J Pharmacol*. 2007;150:873-882
